# Recent Developments in Nanocellulose-Reinforced Rubber Matrix Composites: A Review

**DOI:** 10.3390/polym13040550

**Published:** 2021-02-12

**Authors:** Darren Yi Sern Low, Janarthanan Supramaniam, Apinan Soottitantawat, Tawatchai Charinpanitkul, Wiwut Tanthapanichakoon, Khang Wei Tan, Siah Ying Tang

**Affiliations:** 1 School of Energy and Chemical Engineering, Xiamen University Malaysia, Sepang 43900, Selangor Darul Ehsan, Malaysia; darrenl333.dl@gmail.com; 2 Chemical Engineering Discipline, School of Engineering, Monash University Malaysia, Bandar Sunway 47500, Selangor Darul Ehsan, Malaysia; janarthanan.supramaniam@monash.edu; 3 Center of Excellence in Particle Technology and Materials Processing, Department of Chemical Engineering, Faculty of Engineering, Chulalongkorn University, Bangkok 10330, Thailand; apinan.s@chula.ac.th (A.S.); tawatchai.c@chula.ac.th (T.C.); wiwutt@scg.com (W.T.); 4 Academy of Science, Royal Society of Thailand, Bangkok 10300, Thailand; 5 Advanced Engineering Platform, School of Engineering, Monash University Malaysia, Bandar Sunway 47500, Selangor Darul Ehsan, Malaysia; 6 Tropical Medicine and Biology Platform, School of Science, Monash University Malaysia, Bandar Sunway 47500, Selangor Darul Ehsan, Malaysia

**Keywords:** nanocellulose, rubber, filler, nanocomposites, reinforcement

## Abstract

Research and development of nanocellulose and nanocellulose-reinforced composite materials have garnered substantial interest in recent years. This is greatly attributed to its unique functionalities and properties, such as being renewable, sustainable, possessing high mechanical strengths, having low weight and cost. This review aims to highlight recent developments in incorporating nanocellulose into rubber matrices as a reinforcing filler material. It encompasses an introduction to natural and synthetic rubbers as a commodity at large and conventional fillers used today in rubber processing, such as carbon black and silica. Subsequently, different types of nanocellulose would be addressed, including its common sources, dimensions, and mechanical properties, followed by recent isolation techniques of nanocellulose from its resource and application in rubber reinforcement. The review also gathers recent studies and qualitative findings on the incorporation of a myriad of nanocellulose variants into various types of rubber matrices with the main goal of enhancing its mechanical integrity and potentially phasing out conventional rubber fillers. The mechanism of reinforcement and mechanical behaviors of these nanocomposites are highlighted. This article concludes with potential industrial applications of nanocellulose-reinforced rubber composites and the way forward with this technology.

## 1. Introduction

In recent decades, applications of natural, renewable, environmentally friendly, and sustainable materials have become increasingly important in the fields of polymer science and engineering. It was anticipated that this field of research would excel in producing valuable products with lower carbon footprints and environmental consequences. In this context, the use of organic materials in polymers to improve their properties has garnered much interest from the scientific community and even large-scale industries. Such methods could be a reasonable substitute for non-renewable sources or synthetic chemicals. Out of the myriad of natural materials that could be easily obtained on Earth, cellulose is by far the most abundant renewable organic compound compared to its counterparts such as chitin, alginate and chitosan [1]. Using appropriate methods, cellulose could be isolated from natural sources such as plants, algae, bacteria, and tunicates [2,3]. This compound also has excellent potential and versatility to be modified, functionalized, and tailored to enhance its specific properties for its intended applications. When cellulosic chains are bundled together, they create highly ordered structures (cellulose nanoparticles). These nanoparticles are widely considered as a “green” compound of the future, owing to their unique physicochemical features such as hydrophilicity, chirality, biocompatibility, and biodegradability [2,4]. Other advantages of cellulose and its derivatives include chemical resistance, high stiffness, low-density, dimensional stability, low-cost, non-abrasiveness, and good adaptability to surface modifications through chemical reactions [3,5].

Elastomers, on the other hand, are materials with elastic nature and extensibility attributed to their molecular structure. In general, this class of materials possesses an amorphous structure with a low modulus. Some examples of elastomeric materials include, but are not restricted to, all kinds of rubber produced from natural and synthetic origins, thermoplastics, silicones and ethylene-vinyl acetate [6]. A large subset of this elastomeric materials class is rubbers. Southeast Asian regions dominate rubber-based industries since the 20th-century, such as Malaysia being the past leader as the world’s largest producer and exporter of natural rubber [7]. Presently, the top spot has been taken over by Thailand, followed by Indonesia and Vietnam [7]. Most of the resources such as natural rubber latex, rubber variants and rubber-based products are exported internationally to China, Europe and the United States [7]. The use of rubber has found its place in various industries such as in automotive, machinery, aerospace, adhesives, electrical and electronics, chemicals and ever more-so in healthcare [8]. Global demands for rubber are ever-increasing as its multifunctionality and compatibility are being explored daily in a plethora of applications.

Rubber is considered a large group of polymers that are a subset of elastomers. In this context, the elastomer with the longest history of application is *cis*-1,4-polyisoprene, or more commonly known as the polymer constituent of natural rubber (NR) [9]. NR can be obtained from the milky, slightly dense and viscous latex obtained from lactifier-developing plants of over 2500 species [9,10]. The NR latex is derived from *Hevea brasiliensis* originated from Brazil but is now widely cultivated in Southeast Asia [11]. Other sources of NR latex include *Parthenium argentatum* from Mexico [12], *Taraxacum koksaghyz* from Russia [13], *Solidago altissima* from Canada and the United States, *Eucommia ulmoides* from China [10], as well as *Dyera costulata* from Thailand and Malaysia [14].

**Figure 1:**
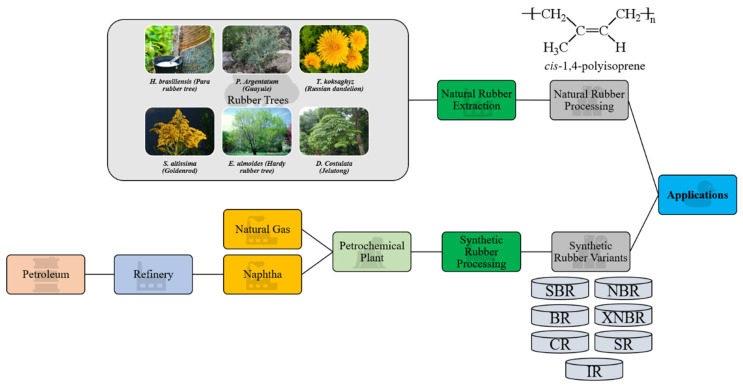
Summarized process chain for natural and synthetic rubber.

Recently, newly emerging commercial products begin to change from using NR to other synthetic materials. However, as an industrial commodity, NR possesses physical and chemical properties that cannot be fully mimicked by synthetic rubber [15]. Nevertheless, the uses and economy of synthetic rubber are also blooming due to its variety and adaptability for numerous applications while competing with its natural counterpart. Generally, synthetic rubbers are manufactured from byproducts of the petroleum industry, fossil fuel resources and natural gas. For example, one of the byproducts, naphtha, is thermally cracked to produce olefinic monomers, subsequently bonded together with the addition of chemicals, and then undergoes the process of polymerization to produce rubber polymers [14]. Common synthetic rubber types include styrene-butadiene rubber (SBR), polybutadiene rubber (BR), chloroprene rubber (CR), acrylonitrile-butadiene rubber (NBR) and its carboxylated variant (XNBR), silicone rubber (SR) and isoprene rubber (IR). Each type of synthetic rubber has different chemical structures and protruding functional groups so that they could be tailored to excel in certain properties. Some synthetic rubbers such as ethylene-propylene rubber possess excellent heat resistance with the ability to withstand service temperatures of approximately 150 ℃, while NBR is reported to have notable swelling resistance towards hydrocarbon oil [16]. Like NR, synthetic rubber has also found its way in a myriad of industries and applications such as in aerospace, footwear, healthcare equipment and devices, personal protective equipment, toys, latex-based paints, sealants, adhesives and belts for mechanical parts. Both NR and synthetic rubber have their own attracting factors. Hence the selection of material greatly depends on the intended application. Figure 1 shows a summarized processing chain for NR and synthetic rubber which could then be employed in various applications.

**Figure 2:**
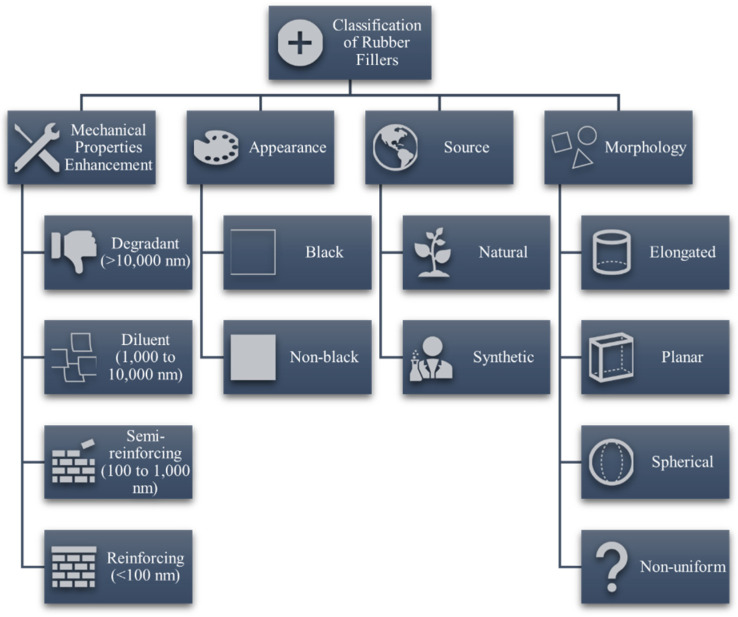
General classification of fillers for rubber sources.

With all the celebration there is with rubber excelling in the global market, it is deemed possible that the agricultural industry could produce sufficient feedstock for both the polymer and filler materials. The use of fillers in vulcanized rubber is a complex subject that is continuously studied by engineers, scientists, and rubber technologists alike. In the process of rubber compounding, various cheaper fillers, such as clay, mica and calcium carbonate, are used to control the final product cost [17]. These fillers are termed “non-reinforcing” because they exert little effect on the mechanical performance of the composite. In addition, such fillers could also affect processing efficiency by significantly changing the manufacturing and functional properties of vulcanizates [18]. On the other hand, some fillers, such as carbon black (CB), silica and talc, are used as reinforcing agents for improving mechanical and strength-related properties such as tensile strength and elasticity before fracture [6,19]. Reinforcing vulcanizates with fillers are important as the former are mechanically weak and difficult to process [20]. A general classification of rubber fillers is depicted in Figure 2. The reinforcing performance of fillers depends greatly on the size and surface area of the particles. As the size of the reinforcing particles decreases, the reinforcing effect increases due to increased effective surface area and improved interfacial interactions with the rubber matrix [21,22,23]. It was categorized that the size ranges smaller than 1000 nm could provide semi-reinforcing effects and those smaller than 100 nm, termed as nanofillers, have more significant reinforcing performance [6,24]. When particle sizes exceed 10,000 nm, which is commonly greater than the polymer interchain distance, the filler could cause regions of delocalized stress, leading to elastomeric chain ruptures during bending, stretching, or flexing.

The use of conventional fillers such as CB and silica has its downsides from an environmental and energy use perspective. Hence there is a need for more sustainable alternatives from nature. The search for substitutes and alternatives of conventional fillers for rubber applications is the driving force in research and development sectors in rubber-based research groups and large-scale industries. Recent advances show the prowess of nanocellulose in achieving or surpassing the current reinforcing performance of conventional fillers [25,26].

Therefore, this review aims to make a summary of recent studies regarding the reinforcement of rubber matrices with various forms of cellulose and their resulting mechanical enhancements. A brief introduction on rubber variants and conventional fillers would be first presented. This would then flow to an introduction to cellulose and its types in terms of structure, size, and sources. Subsequently, recent developments in cellulosic surface modification for applications in rubber reinforcement would be addressed. This would be related to improvements or breakthroughs achieved in terms of mechanical performance using nanocellulose as a filler in rubber. In a separate section, we would shed some light on current developments or future applications of nanocellulose to enlighten readers about the potential of organic fillers as a sustainable reinforcing agent in rubber. As a closing remark, some comments would be provided on the outlook of this innovative technology and how the inclusion of cellulose into the rubber industry could bring mutual benefits to all its beneficiaries.

## 2. Conventional Rubber Fillers for Mechanical Reinforcement

NR is a natural polymer which, after vulcanization (curing), exhibits exceptional properties such as high tensile strength due to its capability in spontaneous crystallization when strained [27,28]. Some other properties such as modulus, abrasion resistance and hardness require assisted improvements for their targeted applications. Conventional particulate fillers for rubber specifically for mechanical reinforcement are mainly represented by two pioneer members, namely carbon black (CB) and silica, where the former was used first in rubber industries. These fillers are commonly used since the 1920s to enhance the mechanical properties of a variety of rubbers [29]. Rubber reinforced with CB would exhibit a higher modulus than silica, but the latter provides a more well-rounded and holistic improvement in tear strength and adhesion properties for a wider range of applications [27].

### 2.1. Carbon Black (CB)

**Figure 3:**
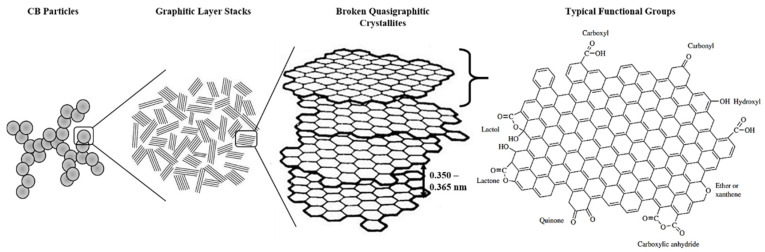
Structure of CB and its typical surface functional groups. Adapted with permission from [36]. Copyright 2003 John Wiley & Sons.

In rubber processing, the addition of CB into rubber compounds aims to strengthen the intermolecular bonds between the compound-forming molecules. Additionally, CB aids in maximizing the volume, improve the physical properties of the rubber and ameliorate vulcanization, owing to its small particle size and possessing multiple functional groups, as shown in Figure 3. Meanwhile, the use of CB reduces the stickiness of the rubber, which may be advantageous in certain applications [30,31]. Nevertheless, CB remains to be one of the pioneer members today and is still widely used in large-scale rubber processing industries.

CB is a carbonaceous material that exists close to its pure form as byproducts from hydrocarbon fuel or biomass processing. It comprises more than 98% of elemental carbon, which consists of spherical carbon atoms that aggregate together [32]. These aggregates can cluster up as agglomerates that break apart during rubber processing and compounding. Diameters of CB particles can range from 10 nm up to 500 nm and vary based on source [19,33]. This variation may be attributed to several factors such as processing combustion temperature and combustion duration. It was also deemed that particle sizes of CB exceeding 1000 nm do not significantly aid rubber reinforcement but may be used to increase latex viscosity caused by hydrodynamic and Payne effects [31,34]. These large sizes lead to a more graphitic structure of CB [19,34]. The variance of this material has led to different types of CB, such as furnace black, thermal black, acetylene black, channel black and lampblack [31]. As CB particles primarily exist in aggregates and not as particulates, their three-dimensional arrangement designates the structure of the CB, which is categorized under ASTM D1765 standard nomenclature. Table 1 shows a collection of typical CB varieties, their mean particle sizes and nitrogen surface areas [19,33,35].

Detailed chemical analysis of rubber-grade CB shows that other elements such as hydrogen, oxygen, nitrogen, and sulfur are also present [36]. The compositions of these elements vary depending on the fuel source and are small but non-negligibly significant. As CB are produced from hydrocarbon fuels, dangling bonds at terminal edges of its graphitic planes, which consist of large polycyclic aromatic rings, are saturated with hydrogen atoms. Oxygen is the common element present in all protruding surface functional groups, and they influence the physicochemical properties of CB, such as chemical interaction reactivity and attachment potential [31]. In the context of rubber processing, CB surface oxidation reduces pH and changes the kinetics of rubber vulcanization. Strong chemical interaction between CB and rubber compounds could contribute in two different ways, namely breaking up agglomerates during the mixing step and preventing particle re-agglomeration. Large amounts of reactive carbon–carbon double bonds, presence of sulfur, olefins and radicals also indirectly help in reinforcing the rubber material cohesively [19]. The anisometric structure of CB aggregates is conducive to create entanglements of rubber polymer chains with CB through mechanical interlocking [31]. When uncured rubber is blended homogeneously with CB, the rubber chains are bound to CB aggregates through several ways, such as physical and chemical interactions, chain immobilization through the creation of glassy-like bridges between filler particles or mechanical interlocking of rubber chains around the filler surface, creating a rubber shell [29,31]. The last mechanism causes the bounded portion of the rubber to be elastically ineffective (occluded rubber) but indirectly increases effective filler volume as rigid particles [29]. In summary, the reinforcing activity of CB is mainly contributed by mechanical interlocking of polymeric chains onto the CB surfaces, chemisorption reactions as well as van der Waals forces between the CB and rubber.

### 2.2. Silica

Following the success of CB, precipitated silica also has found its use as a filler in passenger car tires. The technology for using silica in tires has started since the introduction of “Green Tires” by Michelin in the early 1990s [37]. Replacement of CB with silica was deemed not only to save energy during logistics but also to improve the recycling process of used tires at end-of-life cycles [38]. This technology claims 3% in savings of fuel caused by a 20% drop in rolling resistance compared to CB-filled tires [37]. Advantages of using silica as a reinforcing agent in rubber include substantial mechanical properties, exceptional rolling resistance, high resilience and slow heat buildup [39]. Silica technology could be used directly if the rubber is polar and functionalized. However, in the case of nonpolar and non-functionalized rubber, coupling agents such as silane are required due to polarity differences between the two [37].

From a chemical perspective, the surface of silica is coated with silanol groups, which are polar and chemically reactive. This group leads to difficulty in processing due to low compatibility with hydrocarbon rubbers [29]. Consequently, the filler-rubber interactions would be weak, and silica would not carry out its intended function optimally. Silane coupling agents significantly improve filler-rubber interactions and filler dispersion in the rubber matrix [29,39]. For rubbers filled with silica-silane compounds, silica aggregates form a network mesh trapping occluded rubber through hydrogen bonding. With silane coupling agents, a coating of bound rubber would be formed chemically around the silica aggregates. Simultaneously, some weak silica-rubber interactions may occur due to the surface adsorption of rubber chains on the modified surface. Under deformation, the filler network breaks open and exposes the occluded rubber, causing matrix deformities. However, with the addition of coupling agents, rubber chains attached to the silica surface and occluded rubber remain intact, hence substantiating its mechanical integrity [29]. A study by Majesté and Vincent [40] linked the covering rate of aggregate surfaces by physically adsorbed rubber with reinforcement indicators, showing the evolution of rubber reinforcement with time. It was revealed that there is a replacement of strong filler-filler interactions with weaker filler-rubber ones. Increasing coupling agent content does improve the overall reinforcement index but up to a certain extent. Experiments by Kaewsakul et al. [41] show that chemically bonded rubber contents plateau once silane content in coupling agents approach 10 wt %. Other approaches to improve silica compatibility with rubber include functionalizing rubber compounds with polar groups [42,43], modification of silica surface through grafting [44], combining it with CB [45], making hybrid fillers with graphene nanoplatelets [46] and synthesizing novel silica with high dispersity [47].

## 3. Nanocellulose as Promising Fillers

**Figure 4:**
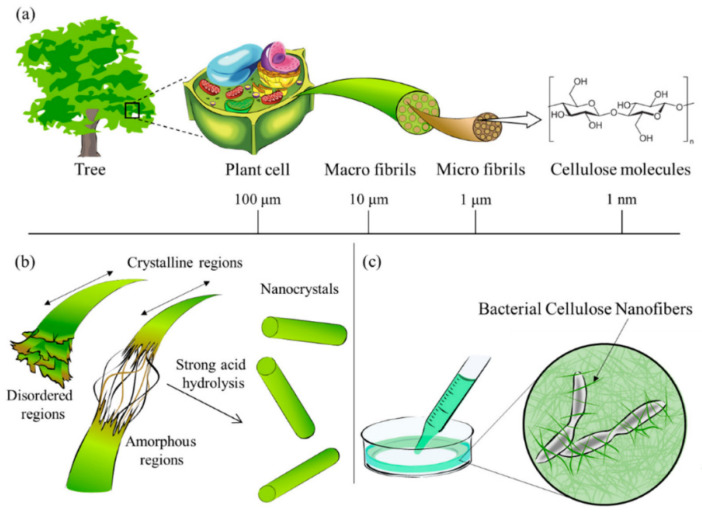
(**a**) Cellulose contained in plants or trees has a hierarchical structure from the meter to the nanometer scale, (**b**) schematic diagram of the reaction between cellulose and strong acid to obtain nanocellulose, (**c**) bionanocellulose cultured from cellulose-synthesizing bacteria. Reprinted from [56].

Cellulose is the most abundant renewable organic material that could be found on Earth [48,49]. From a chemical standpoint, cellulose is a high molecular weight polysaccharide that is constituted from the repetition of 10,000 to 15,000 β-(1,4)-bound-D-glucopyranosyl units [50,51]. These units are arranged in ^4^C_1_-chain configurations where each repeating monomer is rotated 180° to its adjacent unit depending on the source [52,53]. These cellobiose monomer units are linked together to form crystalline structures of cellulose or elementary fibrils. Bundled fibrils produce microfibrils, which in bulk form could lead to macrofibrils or cellulose fibers. Functional groups present along the cellulose chain bestow it with remarkable properties such as chirality, hydrophilicity, adaptability to chemical changes and infusibility. These properties vary depending on the degree of polymerization, cellulose chain length and its source. Cellulose in nature exists both in crystalline and amorphous phases, and the proportion of them highly depends on the source as well as isolation techniques used. Amorphous phases have a lower density compared to crystalline ones and are more prone to react with other molecules. On the other hand, crystalline domains are generally more resistant to mechanical, chemical, biological, and enzymatic treatments [5]. Different isolation techniques, inter- and intramolecular forces, as well as molecular configurations, enable cellulose to exhibit allomorphs such as I_α_, I_β_, II, III_I_, III_II_, IV_I_ and IV_II_ [5,51]. These allomorphs could be transformed from one to another through thermal or chemical reactions [54,55]. Figure 4 shows a hierarchical presentation of cellulose obtained from natural resources from the meter to the nanometer scale.

As cellulose is easily obtainable from various natural sources, it differs widely in terms of type, chain length, morphology, and lignocellulosic content. Within the same source, cellulose maturity, pretreatment, and isolation methods are also contributing factors to the variance in celluloses obtained. A plethora of natural sources such as plants, wood, agricultural crops, animals, algae, and certain bacterial strains can be used to obtain cellulose [57,58]. Table 2 shows a compilation of natural sources of cellulose and its corresponding cellulosic allomorph. Cellulose I_α_ and I_β_ are native allomorphs that always coexist with each other naturally, usually in the same microfibril structure [59]. The allomorphs differ in terms of triclinic and monoclinic arrangements of the cellobiose units. Within and between sheets, these units are linked with hydrogen bonds and van der Waals forces, respectively.

In recent decades, cellulose in the form of nanostructures, also known as nanocellulose (NC), has been proven as one of the most prospective sustainable materials of the future. On top of existing advantages, NC in the nanoscale dimension opens up a new realm of potential applications owing to its higher surface-area-to-volume ratio, high mechanical strength and high Young’s modulus [5]. NC could be classified into three material types, namely cellulose nanocrystals (CNCs), cellulose nanofibers (CNFs) and bacterial cellulose (BC) [69]. There are also articles breaking down NC into two additional types, namely amorphous nanocellulose (ANC) and cellulose nanoyarns (CNY) [70,71]. Processing methods vary depending on the source and type of NC intended to be extracted. Table 3 shows a summary of these types of NC, their sources, extraction methods, dimensions, degree of polymerization and mechanical properties. Different types of NC can exhibit distinct properties which could be further tailored to suit certain specific applications. The suitability of each type of NC greatly depends on the particle type and potential compatibility with host matrices, such as in polymers [71,72,73]. The versatility of NC opens a wide range of applications in a variety of industries, and progress in replacing conventional filler materials is emerging with the advancement of chemical technology [74].

## 4. Nanocellulose Isolation Techniques

**Figure 5:**
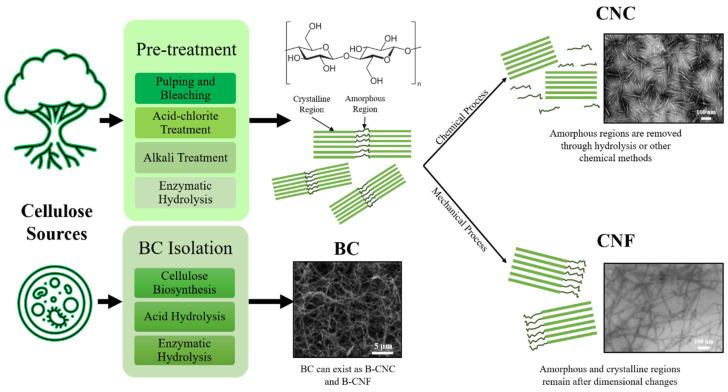
General process flow of isolating cellulose nanocrystals (CNCs), cellulose nanofibers (CNFs) and bacterial celluloses (BCs) from cellulose sources. TEM micrographs adapted with permission from [69]. Copyright 2016 Elsevier.

As most natural sources constitute non-cellulosic components such as lignin, wax, and hemicellulose, the removal of these constituents increases NC purity and eases processability. Usually, NC isolation would involve pretreatment steps to save energy consumption significantly [79,80]. Obviously, saving energy would also mean saving operational costs. Figure 5 shows the summarized process flow to obtain CNCs, CNFs and BCs from NC sources.

To obtain nanostructured cellulose from its natural resource, it is essential to deconstruct the hierarchical structure of cellulose down by at least one order of nanometer scale. Pre-treatment methods are aimed to remove amorphous regions of cellulose and other non-cellulosic compounds. Pulping is a common technique in which cellulose is extracted from woody sources. This technique involves the mechanical and chemical separation of fibers with the aid of solvents such as water, methanol, acetic acid, acetone, and sulfur dioxide [81]. The addition of these solvents reduces energy consumption in the conversion of woody sources to wood pulp. Mechanical means involve equipment such as revolving stone grinders and mills, while chemical processes (i.e., Kraft pulping) use batch digesters and chemicals such as caustic alkali and sodium sulfide to separate lignin and hemicellulose from lignocellulosic biomass [82]. Subsequently, bleaching of wood pulp removes residual non-cellulosic constituents and decolorizes the pulp, leaving cellulose extracts having a white-yellowish color. Some of these decolorants include hydrogen peroxide and sodium dithionate [83]. Cellulose sources could also be treated with diluted acid or base to dissolve hemicellulose and lignin compounds to obtain cellulose fibers. Alkaline compounds, such as sodium hydroxide and calcium hydroxide, as well as acids such as sulfuric acid, are widely used for cellulose pre-treatment due to their availability in large-scale industries [73,81]. Hydrolysis with the use of enzymes could also be an approach in the pre-treatment step, where unique enzymes would degrade lignin and hemicellulose. Although the use of enzymes is more time-consuming, it is more sustainable, environmentally friendly, and a non-energy-intensive technique. Compatible enzymes such as endoglucanses, xylanases, ligninases and cellobiohydrolases are used in this technique [81,84]. On top of covering a plethora of pre-treatment techniques for lignocellulosic biomass, reviews from Kumar and Sharma [85] and Baruah et al. [86] also included biological approaches using an array of fungi such as brown and white fungal species to degrade lignin and hemicellulose. Furthermore, oxidative treatments such as oxidation with 2, 2, 6, 6-tetramethyl piperidinyl oxyl (TEMPO) could be deployed to propel hydroxyl moieties in lignocellulosic compounds into carboxyl groups [79]. When conducted under controlled environments, TEMPO-aided oxidation aids in separating cellulose fibers by repelling carboxyl groups attached to the surface of cellulose structures [73].

Referring to Figure 5, isolation techniques to obtain CNCs and CNFs generally revolve around chemical hydrolysis and mechanical disintegration, respectively [87]. Characteristics of isolated CNCs greatly depend on several hydrolysis parameters such as type of acid, the concentration of acid and duration of hydrolysis. Sulfuric acid is preferred compared to others for hydrolysis (i.e., hydrochloric acid) as it produces negative charge moieties on the NC structure and imparts colloidal stability in water [70,71]. This stability enables the easy processing of NC in water without the requirements of other solvents or chemical reagents [79]. An experiment conducted by Beltramino et al. [88] has shown that using 62 wt.% sulfuric acids at 47 °C for 25 min gave a hydrolysis yield of 72.4 ± 1.2%, which could be further improved by 10% with the use of cellulase enzymes. The use of a certain type of enzyme would depend greatly on the structure of NC desired as the end-product. A review by Houfani et al. [89] explained the synergy between different enzymes in degrading cellulose chains when mixed together. Each enzyme is attracted to a specific section of the cellulose chain and cleaves the polymer bridges linking them together at different regions. For instance, cellobiohydrolases I and II act on the reducing and non-reducing ends of the cellulose chain, respectively, creating cellobiose units. Endoglucanses cleave cellulose chains randomly at the amorphous domains while β-glucosidases hydrolyze cellobiose units into its glucose monomers [89]. Additionally, a new class of enzymes called lytic polysaccharide monooxygenases (LPMO) exhibits synergy with cellulases through redox reactions to increase hydrolysis yield [90]. Another chemical approach for NC isolation is oxidative degradation using TEMPO. The use of TEMPO-oxidative systems (such as TEMPO/NaBr/NaClO in basic conditions and TEMPO NaClO/NaClO_2_ in mildly acidic conditions) could be carried forward from pre-treatment stages to effectively isolate CNCs and CNFs from bulk NC [91]. The use of TEMPO-oxidation does not affect the structural properties of NC and selectively converts C6 primary hydroxyl groups into charged carboxyl groups without affecting secondary hydroxyls [92]. Cellulose fibers could then be separated easily with the repulsion of carboxylate groups. The use of chemical techniques to isolate mainly CNCs and CNFs could be intensified further with the assistance of ultrasonic (US) treatment. The use of US technology has begun to pave its way in industries due to its simple infrastructure, high efficiency, high yield, and ease in scalability [93]. Investigations by Zheng et al. [94] paired US treatment with TEMPO-oxidation as well as sulfuric acid hydrolysis to isolate NC from walnut shells. They found that the use of US energy did not change the integrity of the native cellulose crystals. The use of US treatment was also shown to be productive in dispersing pulp cellulose into CNFs and CNCs after undergoing deep eutectic solvent (DES) pre-treatment processes [95]. The use of US energy is based on cavitation technology in dispersing bulk NC into its subunits and requires optimization in terms of exposure duration and US power. A study by Shojaeiarani et al. [96] showed that a longer US treatment time of 10 min and higher amplitude of 90 µm yielded smaller CNC sizes. The length of CNCs obtained were also 17% shorter when compared to that with smaller amplitudes of 60 µm. However, longer US exposure and higher amplitudes would lead to destroying crystalline structures of CNC and affecting its morphology [96].

To isolate CNFs, the use of mechanical methods was greatly sought after, which does not involve the dissolution of amorphous regions but fibrillating bulk NC. In general, mechanical methods are recognized to be more environmentally friendly without the use of chemical reagents [97]. However, certain approaches may be energy-intensive to achieve nanoscale cellulose structures suitable for their intended applications. A common mechanical approach to fibrillate bulk NC is the use of high-pressure homogenization and micro-fluidization. Both approaches work by passing NC slurries through narrow channels, forming collisions and shear between cellulose fibers at molecular levels [73]. The reduction in the size of fibrillated cellulose could be achieved through large pressure drops, turbulent flow, high shearing forces and interparticle collisions. Additionally, micro-fluidization is a subset of high-pressure homogenization where the difference is that the former operates on the principle of constant shearing rates rather than the continuous application of high-pressures ranging from 50 to 2000 MPa [70]. It involves NC slurries being passed through Z-shaped or Y-shaped chambers where it reaches high velocities and shearing forces for fibrillation. Microfluidizers have a smaller operating pressure range of up to 276 MPa and may require several passes to improve the degree of fibrillation [70]. A paper by Zhuo et al. [97] combined grinding and high-pressure homogenization processes with the aim to reduce overall energy consumption and achieve high NC aspect ratios. This optimization achieved isolated NC with diameters of ~10 nm, lengths greater than 10 µm and aspect ratios higher than 1000. Reports from Ang et al. [98] and Taheri and Samyn [99] coherently concluded that homogenization and microfluidization do generate CNF with high uniformity. However, optimization is required from the perspective of energy consumption and the number of passes, as aggregation could be caused by increased surface areas.

A more direct approach to obtain CNF is by using liquid nitrogen and manual crushing. Cryo-crushing involves the use of a mortar and pestle to crush frozen bulk cellulose. Kargarzadeh et al. [70] explained that this method would yield cellulose fibrils of larger diameters, ranging from 0.1 to 1 µm. Like grinding and milling, cryocrushing could be used as a pre-treatment method before homogenization or micro-fluidization to prevent clogging issues [49]. An electrohydrodynamic approach could also be employed due to its simplicity, cost-efficiency, versatility and scalability [100]. Electrospinning is a technique to isolate CNF through the action of electrostatic forces and potential differences. Bulk NC is pumped through the needle and forms a Taylor cone once the electric potential surpasses the surface tension of the droplet. As voltage increases, the cone elongates and ejects a stream onto the substrate (collector). As the slurry solvent evaporates, the diameter decreases and micro or nanoscale fibers are formed. A recent study by Angel et al. [100] concluded that 12% to 15% (*w*/*v*) cellulose acetate concentration produced the best quality fibers with no “beading” formation through electrospinning. However, they found that the fibers produced were ununiform in diameter with a large range from 404 nm to 1346 nm. It was suggested to use less volatile solutions or co-solvents to produce finer and more uniform fibers [101]. To save energy consumption and improve the quality (i.e., higher CNF percentage by weight), Ho et al. [102] used a twin-screw extruder to fibrillate bulk NC with high solid contents up to 45 wt.%. They have also shown that particle sizes begin to plateau once the number of passes approaches 5, and the degree of crystallinity decreases as the pulp undergoes more passes. In this process, NC slurries are passed through long conveying screws (extruders) comprised of mixing elements and kneading disks that fibrillate bulk NC into CNFs. The process takes place at low temperatures (0 °C to 10 °C), controlled with cooling water circulations to prevent overheating of the kneading area, which may consequently affect the moisture of the pulp [102,103]. It was reported by Rol et al. [103] that CNFs produced ranged from 25 nm to 35 nm depending on the pretreatment method. As previously reported by the same team that the optimal number of passes was 7, recent reports show that there is progress in reducing the optimal number of passes to save energy whilst maintaining CNF quality and strength through simulation and combination of existing extrusion designs [104,105]. As previously mentioned that the use of US energy is compatible and could further improve CNF yield, Debiagi et al. [106] combined US with reactive extrusion to isolate CNF from oat hulls with 60% to 65% yield.

Biosynthesis of BC is closely related to numerous metabolic pathways, each playing its role in synthesizing the biomolecule. Some of these include the Krebs (TCA) cycle, the pentose-phosphate (PP) pathway, gluconeogenesis and the Embden–Meyerhof–Parnas (EMP) pathway [107]. Many compounds and intermediates such as glycerol, pyruvates, hexoses, and dicarboxylic acids can be converted to cellulose compounds. The most efficient BC producer is *Komagataeibacter xylinus (K. xylinus)*, known previously as *Gluconacetobacter xylinus* or *Acetobacter xylinus* [62]. It is a Gram-negative aerobic bacterium. Briefly, BC is biosynthesized by acetic acid bacteria in a culture medium via oxidative fermentation processes. Under suitable conditions for bacterial growth, glucose becomes the source of carbon, peptones for nitrogen, yeast for vitamins and disodium phosphate and citric acid as the phosphate buffer for the culture medium [62]. The biosynthesis of BC by *K. xylinus* involves the process of polymerizing glucose into linear β-(1,4)-glucan chains and has become a model for other NC biosynthesis pathways. A detailed schematic of carbon metabolism and BC biosynthesis pathways of *K. xylinus* was presented by Wang et al. [108] and subsequently summarized into a 21-step network in reviews by Choi and Shin [62] and Jacek et al. [107]. BC could undergo similar isolation techniques like that from plant sources, depending on the dimensions of the intended NC product. One of those is acid hydrolysis using sulfuric acid, hydrochloric acid, or a combination of them. Singhsa et al. [109] experimented with these acids and found that the highest B-CNC yield was obtained from hydrochloric acid (~85%), followed by a sulfuric-hydrochloric acid mixture (~82%) then sulfuric acid (~80%). It was justified that the action of sulfuric acid was too potent and rapidly hydrolyses less ordered regions of the BC chains, separating crystalline domains of the NC [109]. Although the use of sulfuric acid alone for hydrolysis had the lowest relative yield, the resultant crystallite size was the smallest at 6.3 nm for all tested BC sources and was determined to be most stable through zeta potential readings [109]. Furthermore, the use of acids for hydrolysis was deemed to produce BC with lower degrees of polymerization and thermal stability, hence reducing the reinforcing potential of these BCs in polymers [62,110]. Therefore, the use of enzymes for hydrolysis was proposed to be a more feasible technique for isolating BC with lower environmental impacts and yielding better thermal stability and greater mechanical integrity [111,112]. Enzymatic hydrolysis generally involves amorphous domains with large structural faults that render the cleavage of microfibrils into shorter nanocrystals for NC hydrolysis [62]. A report by Domingues et al. [113] made comparisons of hydrolysis processes between acid hydrolysis, enzymatic hydrolysis and a combination of them on eucalyptus fibers. Their results show that acid hydrolysis produced the most stable emulsion with smaller particle sizes. In contrast, the enzymatic route produced unique axial grooves with C-shaped cross-sections and asymmetry during topological analyses [113]. From the perspective of process methodology, it was suggested that a recipe of 2:1 enzyme/BC ratio with reaction time of 30 h or 1:1 enzyme/BC ratio with reaction time of 45 h could result in B-CNC yields of near 25% [110]. However, due to long reaction periods, it was envisaged by the team that the use of surfactants or polyelectrolytes might be necessary to address colloidal stability issues.

## 5. Properties Improvement of Nanocellulose-Reinforced Rubber Composites

**Figure 6:**
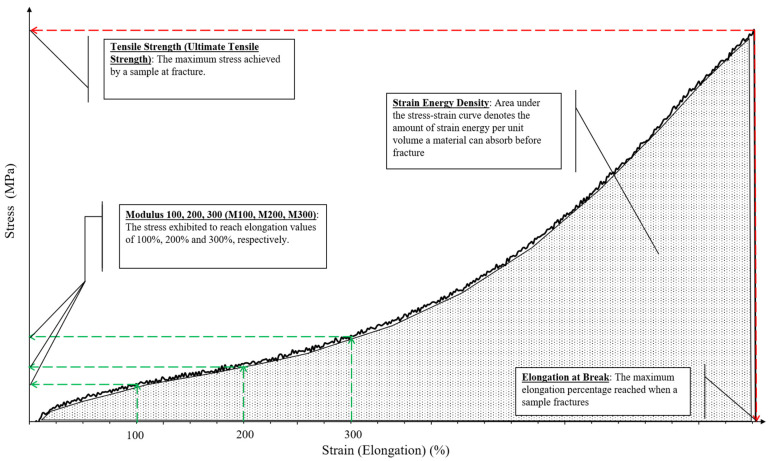
Sample stress–strain curve for rubber materials.

This section would encompass the mechanical improvements achieved through the incorporation of various isolated NCs in rubber polymers. The review would cover a spectrum of natural and synthetic rubber. Mechanical behavior of the rubber, such as tensile strength, elongation at break and Young’s modulus, was mainly emphasized along with strain energy density (SED) from the recent highlighted studies. Briefly, SED or sometimes known as modulus of toughness, is the amount of strain energy that a material can absorb per unit volume before fracture. It is considered as an energetic local way to investigate fatigue failure and fracture in static conditions by making a postulation that brittle fracture happens when the local SED reaches a critical value, known as the critical strain energy [114]. SED values can be determined by mathematical integration to obtain the areas under stress–strain curves, and a larger SED would translate to improved material ductility [114]. The Young’s modulus (gradient of the stress–strain curve in the linear elastic region), on the other hand, may be very small as most elastic regions are short or, in some cases, non-existent or not well defined, especially in rubber materials [115]. Therefore, researchers opt to define “modulus” at a certain strain percentage as a substitute such as M 100, M 200 and M 300. Sample stress–strain curve for a rubber material is shown in Figure 6 with relevant terminologies labeled.

### 5.1. Nanocellulose-Reinforced Natural Rubber Composites

The use of NC from a myriad of sources as a reinforcing agent in NR or blends is comprehensively studied, and the results are tabulated in Table 4. The use of NR as the dispersed aqueous phase is a perfect candidate model system to study the improvement effects of nanofiller reinforcement due to its excellent flexibility [116].

With reference to Table 5, the incorporation of NC from different sources into the NR matrix is similar and facile. NC would be isolated from its natural source through chemical or mechanical means prior to being mixed into the latex. There is no requirement of complex machinery nor elevated operating temperature to blend NC into the matrix. Depending on the scale of sample production, the use of roll mills may be necessary. At a glance, the incorporation of NC into NR has yielded a significant improvement in tensile strength across numerous studies, regardless of NC source and type. Most reports compare samples with and without the presence of NC constituents in vulcanized NR (which include the addition of compounding agents such as accelerators and stabilizers). This indicates that the addition of NC is compatible with existing compounding systems and delivers its function as a reinforcing material [28,117]. Furthermore, a common trend could be observed where elongation at break values decreased, and sample moduli increased. Elongation at break values are related to the ductility of the material, and its decrement could be caused by the agglomeration of NC particles within the rubber matrix [118]. The increase of sample moduli is greatly related to the tensile strength and could be attributed to the restriction of polymer chain movement by the presence of NC particles within the rubber chain network.

Studies from Dominic et al. [74] and Kulshrestha et al. [119] show that partial substitution of CB by CNF of 5 phr and up to 15 phr, respectively, in NR performed substantially well in terms of mechanical strength when compared to systems fully reinforced by CB alone. This suggests that the use of NC could phase out the conventional filler in years to come. In addition, some investigations also involve the addition of foreign reagents to improve interactions between the nanofiller and the rubber matrix, either through surface modification of NCs or adding dispersants in rubber latex. For instance, Cao et al. [120] used carboxylated tunicate CNC in ENR matrices and observed concentration-dependent improvements of crosslink density and an approximate 50% improvement of the tensile strength (from 2.3 MPa to 3.5 MPa) when compared to unmodified tunicate CNC at 5 phr loading. This was attributed to the orientation of modified CNC and rubber chains, which induced stronger interfacial covalent bonds for effective stress transfer at the filler-matrix interfaces. Jiang and Gu [28] added resorcinol-hexamethylenetetramine (RH) into CNCs obtained from four different sources and studied its potential to improve filler-matrix interactions. As a prominent gelation reagent, RH provided good adhesion properties between filler and rubber chains and improved the dispersion of CNCs. This resulted in increased tensile strengths of the nanocomposite of 20% compared to samples without RH. In another study, Parambath Kanoth et al. [121] used free-radical thiol-ene chemistry to modify CNC as an improved nanofiller in NR. The addition of mercapto groups to the CNC doubled the tensile strength when compared to samples without modification, and approximately 5-fold compared to neat NR. The increment in elongation at break also suggests that this functionalization also provided elasticity to the rubber.

**Figure 7:**
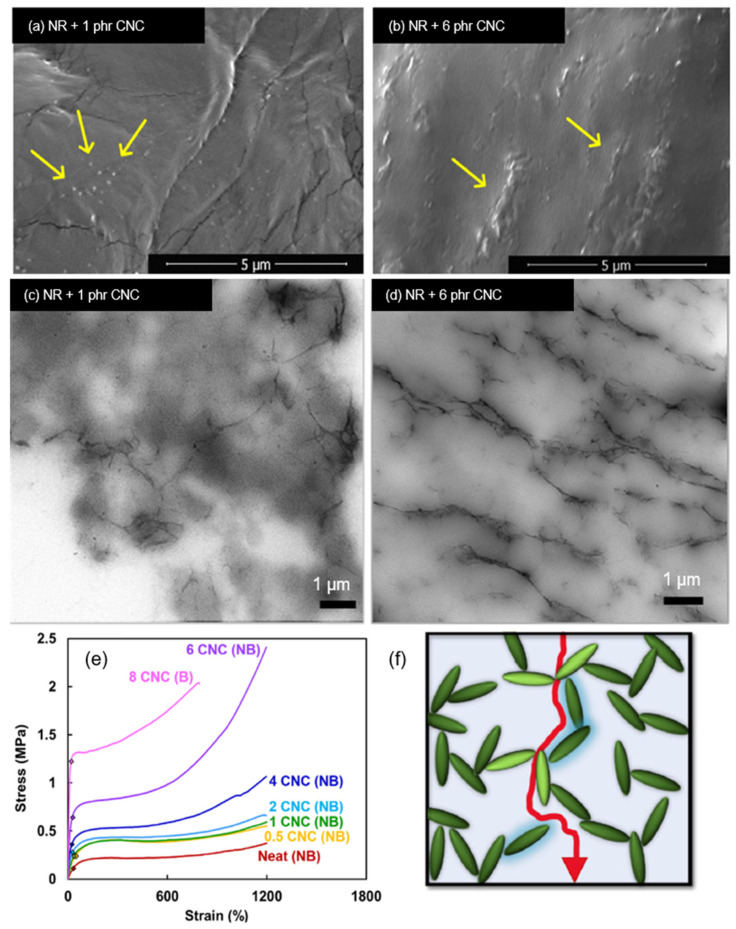
(**a**,**b**) SEM cross-sectional image of natural rubber (NR)/CNC sheets; (**c**,**d**) TEM images showing CNC percolation in formulated rubber thin sheets; (**e**) representative tensile curves for NR/CNC nanocomposite thin sheets, where “NB“ denotes non-broken samples, and “B“ denotes fully broken sample; (**f**) proposed schematic of strong filler–filler interparticle forces as an outcome of percolation effects. Adapted with permission from [122]. Copyright 2020 Elsevier.

**Figure 8:**
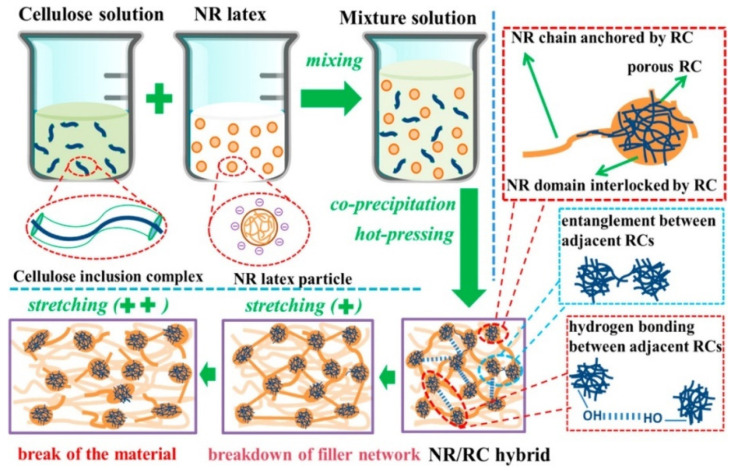
Schematic illustration of NR reinforced with regenerated cellulose in alkaline urea–aqueous system. Reprinted with permission from [123]. Copyright 2017 American Chemical Society.

A comprehensive study by Jardin et al. [122] focuses on the percolation effects of the presence of CNCs in the matrix of natural as well as synthetic rubber. No CNC surface modification was performed, and mutual dispersibility of CNC and rubber latex in water was exploited through thin sheet sample preparation methodologies. Although there were polarity differences between hydrophilic CNC and hydrophobic rubber, the difference in chain structure made agglomeration issues less potent in NR. This was due to the presence of a more pronounced steric hindrance between the CNC and rubber, which limits intimate interfacial interactions between the filler and host matrix [122]. Figure 7a shows specks of CNC (as indicated by the arrows) well-dispersed in the NR latex sheet, whereas Figure 7b shows some percolation of CNC with the formation of CNC networks. Figure 7c,d show that percolation networks are more widely formed with higher concentrations of CNC, which contributes to its greatly improved tensile strength by 8-fold at 6 phr CNC as illustrated in Figure 7e. The formation of percolation effects due to strong filler interactions improves the tear strengths of the sample as it would be more difficult for tearing forces to travel along paths with the least resistance, as shown in Figure 7f. Another investigation by Yu et al. [123] using honeycomb-like structured regenerated nanocellulose (RC) explained that the inclusion of NC in rubber matrices contributes to mechanical improvement in many ways. Referring to Figure 8, hydrodynamic effects arising between the filler and NR, strong interactions due to interlacing phenomena as well, as percolation effects contribute to effective stress distribution, resulting in extraordinary improvements of the tensile strength up to 8.5 times and global modulus up to 29-fold of the nanocomposite at 30 phr RC.

The mechanism of reinforcing NR with the use of an array of NC derivatives is somewhat similar across many studies. In general, the addition of untreated NC into NR latex would reduce mechanical performance due to poor adhesion and agglomeration. The effects of these issues could be minimized or delayed up to higher filler concentrations through surface modification of nanofillers or adding compatibilizers into the rubber latex. However, they could not be eliminated. As previously mentioned, NC derivatives are commonly hydrophilic, whereas rubber matrices are hydrophobic, causing reduced intermolecular interactions between filler and host matrix as well as differences in polarity. Furthermore, when untreated NC compounds are mixed into NR latex, the strain-induced crystallization of NR would be seriously compromised [124]. Agglomeration issues are also common when dealing with NC-rubber nanocomposites. Over a threshold limit of NC fillers present in the rubber matrix, a saturation of the filler becomes visible and causes mechanical performance to deteriorate extensively. From the perspective of CNFs, fiber agglomeration results in the formation of large bundles and interaction between fibers outweigh that of between the fiber and the matrix [124]. As a result, voids would form and become stress concentration points that cause the nanocomposite material to fail prematurely through facilitated crack propagation [122,125]. NC nanofillers are efficacious in strengthening NR nanocomposites through effective stress transfer, which is attributed to their large aspect ratio and exceptional strength independently [28]. At optimal nanofiller concentration and excellent dispersion, NC fillers could interact with each other through hydrogen bonding, which also aids in distributing the stress over the polymer matrix [124]. When stress is applied to the nanocomposite, it is transferred from the host matrix to the nanofillers, which also contributes to a greater tensile modulus. The common trend of reduction in elongation at break values indicates that adding NC nanofillers into NR latex affects its elasticity and stiffness. This value continues to decrease upon the increasing concentration of nanofiller addition and is attributed to the immobilization of rubber polymer chains by well-dispersed nanofiller networks. Incorporation of NC fillers reduces the mobility of the host matrix, resulting in greater stiffness and lower fracture strain values.

### 5.2. Nanocellulose-Reinforced Synthetic Rubber Composites

Alike that of NC-reinforced NR composites, synthetic rubber could also be further reinforced with a variety of NC compounds. However, the issues of dispersibility, agglomeration and interfacial bonding remain while the host polymer network varies. Reports have shown that the strain-induced crystallization phenomenon also occurs in synthetic rubber, similar to that of NR, as the phenomenon is mainly controlled by nucleation processes, which are directly proportional to the strain rate [126]. This greatly depends on the protruding molecular chains along the polymer backbone and its lower stereoregularity [127]. The mechanisms of synthetic rubber reinforcement are comparable to that of its natural counterparts, such as the formation of percolation networks for effective stress transfer through improved interactions between filler molecules as well as between fillers and the host matrix.

When comparing Table 4 and Table 5, it could be observed that there are more studies that involve surface modification of NC fillers prior to addition to the synthetic rubber latex. Synthetic rubber matrices could be selective in their compatibility with surface modifiers, hence opening more opportunities for research and investigations. The application of NC in synthetic rubber is more scarce compared to that in NR as properties of rubber from synthetic origins could be tailored from the formulation stages and may not require additional filler material on top of existing ones. Many studies venture into SBR or IR as the representative synthetic rubber due to its practical significance and a wider range of commercial applications [14]. Scientists are aware of the environmental implications of conventional compounding agents and are exploring routes to reduce the carbon footprint of rubber processing. Hence, using natural and sustainable materials such as NC is a promising solution to the issue.

Xu et al. [128] studied the application of bagasse NC grafted with maleic anhydride (MAH) and styrene as an improvement to neat NC, with prospects of partial substitution of conventional CB filler in SBR matrices. They found that the grafted fillers proved to be more efficient as a reinforcing material through suppression of the Payne effect while increasing modulus and hardness up to a threshold value. The modified NC increased the tensile strength of SBR to a maximum of 32.5 MPa from 30.0 MPa at 10 phr CNC loading, while neat NC showed negative reinforcement potential mostly attributed to agglomeration.

**Figure 9:**
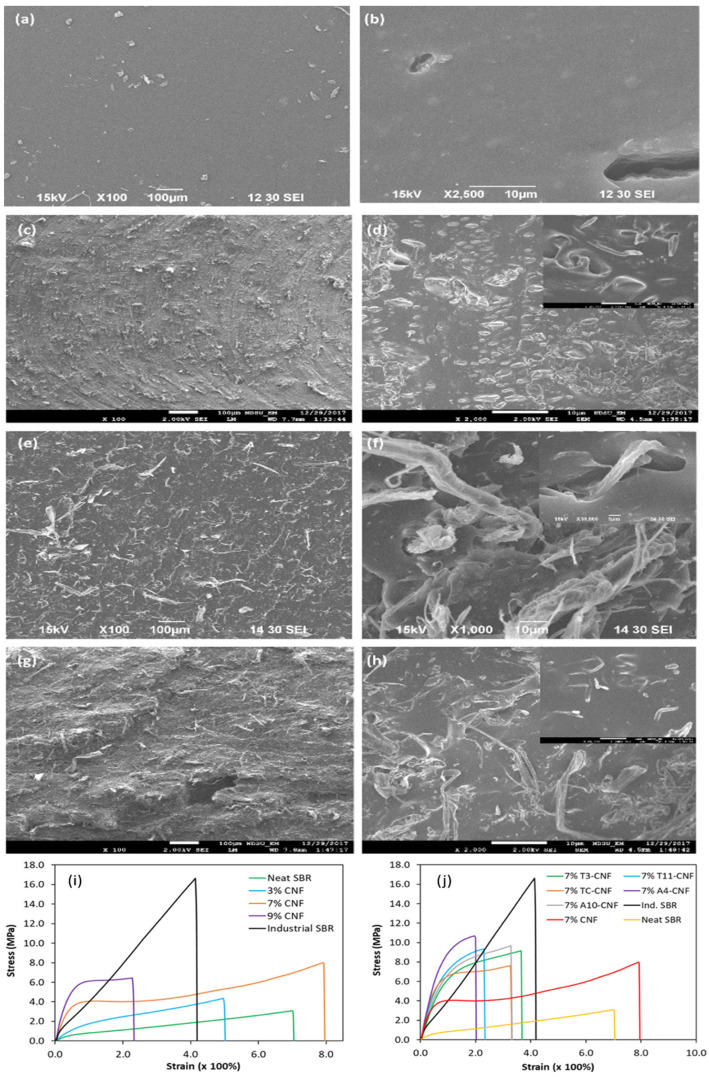
SEM images of tensile fractured surfaces of neat styrene-butadiene rubber (SBR) and SBR/CNFs nanocomposites. (**a**,**b**) Neat SBR; (**c**,**d**) 3% CNFs; (**e**,**f**) 7% CNFs; and (**g**,**h**) 9% CNFs. Representative tensile stress−strain curves of SBR nanocomposites reinforced with (**i**) pristine CNFs and (**j**) various functionalized CNFs. Adapted with permission from [25]. Copyright 2019 American Chemical Society.

In another study, Sinclair et al. [25] explored the potential of CNFs in SBR matrices in terms of nanofiller loading and functional agents. They reported that a moderate concentration of 7 phr CNF managed to improve the tensile strength of neat SBR to about 8 MPa, almost triple the initial value, with significant improvements to Young’s modulus. It was noted that the improvement was contributed by effective load sharing between the CNF and SBR matrix and the formation of a CNF percolation network for efficient stress load distribution. Conversely, a decrease in mechanical strength is common at higher filler loadings, suggesting aggregation of reinforcement agents and formation of defects within the matrix, as depicted in the SEM images in Figure 9a–h. The pristine CNF was functionalized with a plethora of functional agents such as 3-mercaptopropanoic acid (T3), 11-mercaptoundecanoic acid (T11), 4-pentanoic acid (A4), 10-undecenoic acid (A10) and cysteine (TC). Based on Figure 9j, the use of functionalized CNF managed to mimic the stress–strain curve patterns of industrial SBR, which has high tensile strength and moderate strain. CNFs functionalized with mercapto groups (TC-CNF, T3-CNF, and T11-CNF) yielded comparable results despite their difference in chain lengths. This was similar to vinyl-functionalized CNF (A4-CNF and A10-CNF). When compared between groups, A4-CNF provided more significant improvements to SBR in terms of strength (10.5 MPa against 9.4 MPa) and modulus (12.6 MPa against 9.7 MPa) at 7% CNF concentration. These findings indicate that vinyl groups have greater hydrophilic reduction abilities in CNF, which improved the linkages between CNF and SBR during vulcanization.

Fumagalli et al. [26] researched the potential of surface-modified CNCs and CNFs with 3,3′-dithiodipropionic acid chloride (DTACl) in SBR. They reported the occurrence of a reactive interface and strong stiffening behavior with the addition of the modified nanofillers. Impressively, 10 wt% of DTACl-modified CNC and CNF managed to improve the tensile strength of neat SBR by 7-fold and 5-fold, respectively. Other properties such as modulus and elongation at break increased as well, making them comparable to characteristics of rubber reinforced by industrial CB and silica fillers. Furthermore, a study by Wang et al. [129] used bacterial cellulose whiskers without modifications in XNBR and found that the reinforcing potential peaked at 13 phr, providing quadruple improvement in terms of tensile strength, while elongation at break decreased slightly. The tear strength of the sample was also doubled. This was attributed to facile stress transfer through H-bonds within the percolating bacterial nanocellulose network.

## 6. Potential Applications of Nanocellulose-Reinforced Rubber Composites

Recently, naturally derived nanomaterials are playing a crucial role in various fields, including wearables, transport, and biomedical science. However, challenges such as ease of purification methods, mass production and their practical applications remain major concerns [164]. Despite these challenges, some studies involving NC-reinforced rubber composites managed to show notable results. For instance, Nagatani [165] innovated a sponge-rubber material based on NR/CNF composites for sports shoe sole applications. Crosslinkers, such as dicumyl peroxide, were used to produce the material with azodicarbonamide as a chemical blowing agent. The CNF surface modification through oleoylation had greater reinforcing effects and endowed it with hydrophobic properties. The presence of double bonds on the functionalized side chains of the CNF can form crosslinks by reacting with sulfur in rubber compounds [165]. Based on the positive outcome, a sports shoe sole was developed consisting of the composite CNF whose matrix consisted of a blend of ethylene-vinyl acetate copolymer and NR. This robust material is under development to further exploit its benefits and improve the current prototype model of the lightweight CNF-reinforced shoes [165].

Similarly, studies by Visakh et al. [166] and Abraham et al. [125] showed the potential uses of NC-reinforced NR for barrier membrane applications. In the former investigation, the nanocomposites were prepared with crosslinking agents, activators, accelerators, and a set amount of NC dispersion through ball milling and ultrasonication techniques. It was hypothesized that the formation of a zinc-cellulose network complex between ZnO (as an activator) and NC coexisted with the crosslinked NR network. The polarity of the cellulose molecules enabled a strong interconnecting network within the composite structure. As a result, the NC-reinforced NR composite exhibited reduced solvent absorption rates against benzene, toluene, and *p*-xylene [125,166]. The diffusion coefficient also had a decreasing trend against increasing NC concentration. It was proposed that, compared to CNC, the separation efficiency was more efficient when CNF was incorporated into NR due to tangling effects of the nanofibers [125]. These studies show the potential use of NC-reinforced rubber as a membrane barrier material for the separation of organic solvents in addition to enhancing its mechanical integrity.

Another novel application of NC-reinforced rubber is in the field of electronics and wearable sensors. In this context, a report by Silva et al. [167] highlighted the potential use of functionalized CNF/PANI and NR nanocomposite materials in terms of mechanical properties and electrical conductivity. Briefly, CNF was coated with PANI through in-situ polymerization prior to incorporation into the NR matrix. Samples from the study showed that the addition of unfunctionalized CNF into NR improved the tensile strength by 4-fold and functionalized CNF/PANI by more than double. This could be explained by the greater hydrophobicity of CNF compared to CNF/PANI, which results in improved adhesion to the NR polymer chains [167]. Furthermore, upon testing the samples for their electrical conductivity for wearable sensor applications, it was found that the addition of functionalized CNF/PANI in NR increased conductivity of the material as compared to unfunctionalized CNF by about 10-fold [167]. The presence of PANI chains enables the hopping of free charge carriers, which translates to electrical signal conductivity. Thus, the addition of NC into a rubber not only strengthens the polymer matrix but could also be functionalized to endow the material with electrical properties for wearable electronics.

Separately, another study by Phomrak et al. [153] formulated NR latex foam reinforced with BC and NC. In their study, NC was initially dispersed in water, followed by thorough mixing in NR latex. Potassium oleate soap was added to the mixture to make foam until the volume was tripled. Other compounding agents like accelerators, gelling agents and antioxidants were then added and homogenized. The composite porous foams fabricated with the Dunlop method showed increasing trends of tensile strength up to 15 phr of NC addition. Furthermore, with the aim of the composite material to be a sustainable shock absorber or supporting material, compressibility tests were also conducted. It was highlighted that the addition of NC in the NR latex foam also enhances compression recovery of up to 35% due to enhanced adhesion and molecular interactions between NC and NR. Samples in this study showed insignificant effects on thermal stability regardless of the concentration of NC addition, hence drawing the conclusion that the NC-reinforced foam can be used for applications at high temperatures up to 300 °C [153]. These studies show the successes of ongoing investigations regarding applications of nanocellulose-reinforced rubber materials. To ensure continued success, close research communications between the industries and research institutions are essential to make these material innovations feasible and affordable globally.

## 7. Conclusions and Future Outlook

This review encompasses the recent advances of incorporating NC into various rubber matrices and its mechanical integrity improvement. As the demand for safe and less toxic nanofillers for rubber applications surges, it is postulated that natural material will drive industrial needs emphasizing sustainable solutions with goals of replacing harmful nanofillers. NC from a myriad of sources finds use in rubber engineering since they are renewable, sustainable, abundantly available, biodegradable, and low in cost. However, concerns have been highlighted regarding the utilization of organic solvents for the isolation of NC. It is crucial to overcome this problem by using green solvents and venturing into more economical methods for extraction. The issue of hydrophilicity is another impeding factor for widespread use of NC and surface functionalization; hence adding compatibilizers is a promising strategy to supplement it. The use of NC in rubber processing could further proceed with various prototype end-products to prove that this new technology is sustainable without compromising mechanical integrity. Overall, there is a positive outcome from adding NC into rubber matrices, both NR and synthetic rubber, as a filler for reinforcement with optimum amounts. Extensive pools of literature showing success in incorporating NC in rubber processing as an approach of reinforcement have revealed hopeful prospects that are yet to be widely explored. There is great potential to translate these results into applications and products in industries for large-scale production. Rather than keeping concepts of sustainability as a theory, the scientific community should pave the way in inculcating an out-of-the-box mindset to create green rubber materials for the global community.

## Figures and Tables

**Table 1:** Typical rubber grade carbon black (CB) with average particle sizes and nitrogen surface areas.

Name and Abbreviation	ASTM Nomenclature ^1^	Average Particle Size (nm)	Average Nitrogen Surface Area (m^2^/g)
Super abrasion furnace (SAF)	N 110	15–18	124–130
	N 121	17–19	121–122
Intermediate SAF (ISAF)	N 220	20–25	112–115
	N 234	20–26	116–121
High abrasion furnace (HAF)	N 330	28–36	76–80
	N 339	26–34	89–92
Fast extrusion furnace (FEF)	N 550	39–55	39–41
General-purpose furnace (GPF)	N 660	56–70	34–36
Semi-reinforcing furnace—high structure (SFR)	N 774	77–82	28–32
Fine thermal (FT)	N 880	180–200	17–20
Medium thermal (MT)	N 990	250–350	7–9

^1^ The letter N indicates furnace and thermal blacks under “normal curing” conditions.

**Table 2:** General sources of cellulose.

Source	Predominant Allomorph	Elaboration	Ref.
Algae	I_α_ (one-chain triclinic)	Green, yellow, brown, and red algae are suitable sources. Cellulose extracted from the *Cladophorales* order is usually high in crystallinity up to greater than 90%.	[2,60,61]
Bacteria	I_α_ (one-chain triclinic)	Bacterial cellulose (BC) could be extracted from an array of species, out of which the most common is *Komagataeibacter xylinus*. Others include *Salmonella*, *Rhizobium*, *Azerobacter*, *Azotobacter* and *Pseudomonas*. BC has several advantages, such as being mechanically stable, high purity, excellent permeability, non-cytotoxic and good biocompatibility.	[62,63,64]
Plants and agricultural biomass	I_β_ (two-chain monoclinic)	Plants of all varieties and wood have high cellulose content. These resources are widely available in nature, renewable and cost-efficient. Some large-scale crops include oil palm, wheat, rice, coconut husks and bagasse. Some examples of wood sources are eucalyptus, oak, pine, juniper, and cedar.	[65,66]
Tunicates	I_β_ (two-chain monoclinic)	Tunicates are marine invertebrate animals. Skeletal structures of tunicates are made of tunic tissues, which are the main source of cellulose. Tunicate cellulose possesses characteristics such as high aspect ratio and high crystallinity. Some examples include *Ciona intestinalis*, *Styela clava* and *Halocynthia roretzi*.	[67,68]

**Table 3:** Comparison between general types of nanocellulose.

NC Type	Synonyms	Sources	Extraction Methods	Dimensions (L: length, D: diameter)	Cryst. and DP ^1^	Mechanical Properties ^2^	Ref.
CNC	Nano- crystalline cellulose (NCC), cellulose nanowhiskers (CNW), cellulose whiskers, rod-shaped cellulose microcrystals, microcrystalline cellulose, cellulose nanorods	Wood, cotton, sisal, flax, oil palm empty fruit bunches, wheat, algae, rice straw	Acid hydrolysis	L: 70–300 nm D: 3–70 nm	High (~90%), 500 to 15,000	TS: 7500 MPa YM: 100–140 GPa	[2,53,69,73]
CNF	Cellulose nanofibrils, nanofibrillated cellulose (NFC), microfibrillated cellulose (MFC), cellulose microfibrils	Wood, hemp, oil palm empty fruit bunches, flax, cassava potato, bamboo	Mechanical treatments with chemicals or enzymatic treatments	L: a few microns D: 2–20 nm	Large range from 40% to 80%, ≥500	TS: 72.6 ± 7.4 MPa YM: 10.2 ± 1.2 GPa	[73,75,76,77]
BC	Bacterial nanocellulose (BNC), microbial cellulose, biocellulose	Nutritional media such as saccharides and alcohols	Biosynthesis processes	L: a few microns D: 20–100 nm	79% to 92%, 4000 to 10,000	TS: 200–300 MPa YM: 15–35 GPa	[62,78,79]

^1^ Cryst. indicates percentage crystallinity, and DP indicates the degree of polymerization. ^2^ TS stands for tensile strength, and YM represents Young’s modulus.

**Table 4:** Recent reports on nanocellulose (NC)-reinforced natural rubber nanocomposites.

NC Type	NC Source	NC Isolation	NC Surface Modification/Added Compatibilizer	Polymer	Method of Incorporation	Reference Sample ^1^	Mechanical Property Change *					Ref.
							Tensile Strength (MPa)	Elongation at Break (%)	Modulus ^2^ (MPa)		Stress Energy Density (MJ/m^3^)	
CNC	Softwood pulp	Double oxidation with (NH_4_)_2_S_2_O_8_and H_2_O_2_	n/a	NR	RT mixing using two-roll mill	10 phr CNC	From 12.6 ± 0.5 to 24.8 ± 3.7	From 564 ± 10 to 532 ± 30	M 100	From 0.9 ± 0.2 to 1.8 ± 0.4	Not reported	[28]
			RH dispersant in NR			10 phr CNC + RH	From 12.6 ± 0.5 to 33.7 ± 2.5	From 564 ± 10 to 603 ± 21		From 0.9 ± 0.2 to 1.8 ± 0.3		
	Bagasse		n/a			10 phr CNC	From 12.6 ± 0.5 to 27.2 ± 2.9	From 564 ± 10 to 569 ± 12		From 0.9 ± 0.2 to 2.0 ± 0.2		
			RH dispersant in NR			10 phr CNC + RH	From 12.6 ± 0.5 to 30.5 ± 3.5	From 564 ± 10 to 517 ± 10		From 0.9 ± 0.2 to 2.7 ± 0.5		
	Cotton straw		n/a			10 phr CNC	From 12.6 ± 0.5 to 28.1 ± 2.6	From 564 ± 10 to 579 ± 30		From 0.9 ± 0.2 to 1.9 ± 0.2		
			RH dispersant in NR			10 phr CNC + RH	From 12.6 ± 0.5 to 30.4 ± 2.9	From 564 ± 10 to 550 ± 18		From 0.9 ± 0.2 to 2.3 ± 0.3		
	MCC		n/a			10 phr CNC	From 12.6 ± 0.5 to 30.7 ± 1.5	From 564 ± 10 to 575 ± 20		From 0.9 ± 0.2 to 2.2 ± 0.2		
			RH dispersant in NR			10 phr CNC + RH	From 12.6 ± 0.5 to 35.9 ± 5.0	From 564 ± 10 to 575 ± 40		From 0.9 ± 0.2 to 2.6 ± 0.4		
CNC	Soy hulls	H_2_SO_4_ hydrolysis	n/a	NR	RT mechanical mixing	5 dry wt % CNC	From 0.59 ± 0.08 to 3.03 ± 0.11	From 611 ± 71 to 552 ± 9	YM	From 0.6 ± 0.1 to 18.1 ± 2.8	From 2.78 ± 0.43 to 10.74 ± 0.62	[116]
CNC	Tunicate	H_2_SO_4_ hydrolysis	Carboxylated CNC	ENR	RT mechanical mixing	10 phr m-CNC	From 1.29 ± 0.07 to 4.66 ± 0.18	From 493 ± 35 to 522 ± 29	M	From 0.83 ± 0.05 to 3.63 ± 0.15	From 0.402 ± 0.018 to 1.248 ± 0.049	[120]
CNC	Cotton	H_2_SO_4_ hydrolysis	n/a	NR	RT mechanical mixing	10 wt % CNC	From 2.4 ± 0.4 to 4.2 ± 0.8	From 910 ± 174 to 750 ± 125	M5	From 1.01 ± 0.08 to 1.75 ± 0.38	From 1.45 ± 0.41 to 1.56 ± 0.32	[121]
			Surface-modified CNC with thiol groups			10 wt % m-CNC	From 2.4 ± 0.4 to 10.2 ± 1.3	From 910 ± 174 to 1210 ± 110		From 1.01 ± 0.08 to 1.86 ± 0.12	From 1.45 ± 0.41 to 4.60 ± 0.57	
CNC	n/a	H_2_SO_4_ hydrolysis	n/a	NR	RT mechanical mixing	6 phr CNC	From 0.30 to 2.45	Not reported	YM	From 0.2 to 3.8	Not reported	[122]
CNC	Potato starch	H_2_SO_4_ hydrolysis	n/a	NR	RT mechanical mixing	20 dry wt % CNC	From 2.5 to 13.5	From 1351 to 536	M	From 1.0 to 25.0	Not reported	[130]
CNC	Sisal leaves	H_2_SO_4_ hydrolysis	n/a	NR	Hand layup technique	10 wt % CNC	From 0.406 to 0.550	From 352.30 to 312.42	-	Not reported	Not reported	[131]
CNC	Softwood pulp	Double oxidation with (NH_4_)_2_S_2_O_8_and H_2_O_2_	n/a	NR	RT mixing using a two-roll mill	20 phr CNC	From 13.0 to 31.0	From 558.7 to 575	-	Not reported	Not reported	[132]
			Surface-modified CNC with CTMAB surfactant			10 phr m-CNC	From 13.0 to 30.3	From 558.7 to 670.4				
CNC	n/a	Without treatment	Compound subjected to electron beam irradiation	NR	RT mechanical mixing	2 wt % CNC	From 12.30 ± 0.27 to 16.06 ± 1.17	From 723.95 ± 10.54 to 798.02 ± 14.96	M 200	From 0.86 ± 0.04 to 0.95 ± 0.07	Not reported	[133]
	Dried rubber tree leaves	H_2_SO_4_ hydrolysis				2 wt % r-CNC	From 12.30 ± 0.27 to 15.04 ± 1.35	From 723.95 ± 10.54 to 807.23 ± 15.79		From 0.86 ± 0.04 to 1.04 ± 0.07		
CNC	Kraft pulp	H_2_SO_4_ hydrolysis	Formic acid coagulant	NR	RT mixing and homogenization	20 phr CNC	From 7.9 to 17.0	From 520 to 345	M 300	From 2.5 to 6.9	Not reported	[134]
CNC	Bamboo pulp	H_2_SO_4_ hydrolysis	n/a	NR	RT mixing using two-roll mill	10 wt % CNC	From 9.2 ± 1.3 to 17.3 ± 1.4	From 554 ± 9 to 455 ± 11	YM	From 1.7 ± 0.2 to 3.8 ± 0.2	Not reported	[135]
CNC	n/a	Without treatment	n/a	NR	RT mechanical mixing	5 phr CNC	From 2.25 to 7.00	From 610 to 550	M 100	From 1.0 to 6.2	Not reported	[136]
CNC	n/a	Without treatment	Oxidized NR	NR	RT mechanical mixing	5 wt % CNC, second degree of oxidation	From 1.72 ± 0.39 to 2.37 ± 0.42	From 878 ± 57 to 703 ± 43	M	From 1.33 ± 0.39 to 7.92 ± 1.02	Not reported	[137]
CNC	n/a	Without treatment	n/a	NR	RT mixing using two-roll mixing mill	5 phr CNC	From 13.5 ± 0.47 to 15.4 ± 0.58	From 1066 ± 48.7 to 1257 ± 89.5	M 100	From 0.721 ± 0.028 to 0.745 ± 0.025	Not reported	[138]
			Maleated NR as a compatibilizer			5 phr CNC + 10 phr m-NR	From 13.5 ± 0.47 to 21.9 ± 0.84	From 1066 ± 48.7 to 1412 ± 55.5		From 0.721 ± 0.028 to 1.069 ± 0.036		
CNC	Tunicate	Bleaching, H_2_SO_4_ hydrolysis	n/a	ENR	RT mechanical mixing, two-roll mill	10 phr CNC	From 14.4 ± 1.2 to 22.6 ± 1.6	From 674 ± 30 to 474 ± 60	M	From 4.2 ± 0.6 to 10.7 ± 1.1	From 20.4 ± 3.7 to 55.9 ± 5.8	[139]
CNC	Tunicate	H_2_SO_4_ hydrolysis	n/a	ENR	RT mechanical mixing, two-roll mill	20 phr CNC	From 1.15 ± 0.08 to 4.04 ± 0.18	From 306 ± 15 to 198 ± 12	M 100	From 0.59 ± 0.04 to 2.21 ± 0.09	From 2.2 to 4.4	[140]
CNF	Rice husk	Alkali treatment, steam explosion	Partial substitute of CB	NR	RT mixing using two-roll mill	5 wt % CNF + 25 wt % CB	From 22.35 ± 0.44 to 23.74 ± 0.14	From 820 ± 10 to 574 ± 14	M 100	From 0.90 ± 0.08 to 1.98 ± 0.08	Not reported	[74]
CNF	Softwood Kraft pulp	Refiner, Grinder	n/a	NR	RT mixing, kneaded with three-roll mill	5 wt % CNF	From 16.1 ±1.4 to 30.3 ± 0.4	From 623 ± 14 to 718 ± 6	YM	From 1.7 ± 0.0 to 4.4 ± 0.1	Not reported	[117]
			CNF/Stearic acid			5 wt % st-CNF	From 16.1 ± 1.4 to 28.9 ± 1.4	From 623 ± 14 to 530 ± 30		From 1.7 ± 0.0 to 18.3 ± 1.0		
			CNF/Oleic acid			5 wt % ol-CNF	From 16.1 ± 1.4 to 25.6 ± 1.0	From 623 ± 14 to 492 ± 12		From 1.7 ± 0.0 to 12.7 ± 1.9		
CNF	Wheat straw	Alkaline/ acid hydrolysis, US treatment	Partial substitute of CB	NR	RT mixing using two-roll mill	2 phr CNF + 50 phr CB	From 26.0 to 30.1	From 465 to 501	M 300	From 12.5 to 16.5	From 119.0 to 150.8	[119]
CNF	*Cuscuta reflexa* (parasitic plant)	Alkali treatment, steam explosion	n/a	NR	RT mixing using two-roll mill	2 phr CNF	From 20.38 ± 0.44 to 22.78 ± 0.52	From 810 ± 0 to 799 ± 11	M 300	From 2.11 ± 0.04 to 2.70 ± 0.05	Not reported	[124]
CNF	n/a	Without treatment	CNF decorated with ZnO	NR	Dry blending using a two-roll mill	7.5 phr m-CNF (Medium)	From 26.59 ± 0.78 to 26.97 ± 0.59	From 685 ± 9 to 665 ± 4	M 300	From 2.31 ± 0.01 to 2.90 ± 0.01	Not reported	[141]
					Wet blending with a mixer	1 phr m-CNF (Long)	From 26.59 ± 0.78 to 27.49 ± 0.52	From 685 ± 9 to 638 ± 3		From 2.31 ± 0.01 to 4.42 ± 0.05		
CNF	*Agave angustifolia*	Bleaching	n/a	NR/PLA	RT mechanical mixing	7.5 wt % CNF	From 10.4 to 13.0	Not reported	YM	From 1.55 GPa to 1.75 GPa	Not reported	[142]
CNF	Kraft pulp	Disk milling	n/a	NR	RT mechanical mixing	5 phr CNF	From 0.79 to 7.03	From 603 to 508	M 100	From 1.12 to 4.92	Not reported	[143]
CNF	Eucalyptus Kraft pulp	TEMPO- oxidation, Micro- fluidizer	n/a	NR	RT mechanical mixing	10 wt % CNF	From 8.3 ± 0.2 to 18.7 ± 0.4	From 908 ± 13 to 7 ± 0.7	-	Not reported	From 18.1 ± 1.1 to 0.9 ± 0.1 J/m^3^	[144]
CNF	Wood pulp	H_2_SO_4_ hydrolysis	CNF–PANI complex	NR	Demulsification and co- precipitation	~0.60 wt % CNF + 20 wt % PANI	From 1.0 ± 0.1 to 9.7 ± 0.9	From 352 ± 46 to 253 ± 74	YM	From 0.9 ± 0.2 to 10.9 ± 0.9	Not reported	[145]
CNF	Wood fibers	Alkali treatment	n/a	NR	RT mechanical mixing	1 phr CNF	From 11.8 to 12.5	Not reported	-	Not reported	Not reported	[146]
		Alkali treatment, Ultra- sonication	n/a			1 phr u-CNF	From 11.8 to 14.0					
		Alkali treatment, Ultra- sonication	ZnO/CNF hybrid filler			1 phr ZnO/ u-CNF	From 11.8 to 16.3					
CNF	Jute fibers	Alkali treatment, steam explosion	n/a	NR	Ball milling, US treatment	3 wt % CNF	From 3.52 to 4.25	From 860 to 410	-	Not reported	Not reported	[147]
CNF	Jute fibers	Nitro- oxidation	Modified carboxycellulose nanofibers	NR	RT mechanical mixing, sonication	0.4 wt % NO-CNF	From 0.77 to 6.20	From 234 to 3.5	YM	From 3.3 kPa to 1770 kPa	Not reported	[148]
CNF	Spinifex grass	NaOH treatment, HP homogenization	n/a	NR	RT mechanical mixing	0.1 wt % NaOH-CNF	From 24.32 to 25.67	From 1925.2 to 1859.1	-	Not reported	From 125 ± 14 to 131 ± 12	[149]
		Bleaching, HP homogenization				0.1 wt % B-CNF	From 24.32 to 23.69	From 1925.2 to 1882.2			From 125 ± 14 to 127 ± 3	
		Choline chloride- urea treatment, HP homogenization				0.1 wt % CCU-CNF	From 24.32 to 28.45	From 1925.2 to 1920.6			From 125 ± 14 to 142 ± 12	
	Wood pulp	Ultrafine grinding				0.1 wt % M-CNF	From 24.32 to 20.05	From 1925.2 to 1585.9			From 125 ± 14 to 103 ± 11	
BC	*Acetobacter xylinum*	Without treatment	n/a	NR	NR immersion of BC pellicles	2.5 DRC with BC, 50 °C	From 0.8–1.2 to 392	From 100–111 to 3.2	YM	From 1.6–2.4 to ~20,000	Not reported	[150]
BC	*Acetobacter xylinum*	Bio- synthesis	n/a	NR	RT mechanical mixing	10 phr BC	From 20 to 24	From 840 to 750	M 100	From 0.55 to 0.65	Not reported	[151]
BC	Modified Hestrin Shran culture medium	Bio- synthesis	n/a	NR	RT mechanical mixing	10 phr BC	From ~3.0 to ~10.5	From ~875 to ~20	YM	From 0.020 to 0.625	Not reported	[152]
			BC decorated with polystyrene			7 phr d-BC	From ~3.0 to ~6.0	From ~875 to ~25		From 0.02 to 0.41		
BC	n/a	Crushing, homogenization, ball milling	n/a	NR Foam	RT beater homogenization	15 phr BC	From 0.30 to 0.73	From 150 to 100	M 100	From 0.25 to 0.70	Not reported	[153]
BC	n/a	Without treatment	n/a	NR	RT mechanical mixing	80 wt % BC	From 0.8 ± 0.1 to 75.1 ± 27.1	From 111.5 ± 6.4 to 4.3 ± 1.4	YM	From 1.6 ± 0.4 to 4128.4 ± 998.3	Not reported	[154]
BC	n/a	Without treatment	n/a	NR	RT mechanical mixing	80 wt % BC	Up to ~125	Approximately 7	YM	Approximately 4750	Not reported	[155]
			Lactic acid-modified composite			80 wt % BC + 20 wt % a-NR	Up to ~155	Approximately 9		Approximately 6000		

^1^ Reference samples to represent the studies are those that provide the greatest improvement in tensile strength. Elongation at break, modulus, and strain energy density values correspond to that sample. ^2^ The types of moduli reported were classified accordingly as M (not explicitly defined), YM (Young’s modulus) and Mx (where x is a specific strain value). * Standard deviation and significant digits are reported as-is. Value approximations are made if data charts are provided without numerical values.

**Table 5:** Recent reports on NC-reinforced synthetic rubber nanocomposites.

NC Type	NC Source	NC Isolation	NC Surface Modification/ Added Compatibilizer	Polymer	Method of Incorporation	Reference Sample ^1^	Mechanical Property Change **					Ref.
							Tensile Strength (MPa)	Elongation at Break (%)	Modulus ^2^ (MPa)		Strain Energy Density (MJ/m^3^)	
CNC	Cotton linter	H_2_SO_4_ hydrolysis	CNC surface modified with DTACl	SBR	Mechanical mixing at 100 °C	10 wt % m-CNC	From 10 ± 1 to 70 ± 5	From 368 ± 25 to 427 ± 21	YM	From 2.0 ± 0.1 to 6.6 ± 0.2	Not reported	[26]
CNC	n/a	H_2_SO_4_ hydrolysis	n/a	SBR	RT mechanical mixing	2 phr CNC	From 0.85 to 1.50	Not reported	YM	From 2.8 to 5.0	Not reported	[122]
CNC	Bagasse	Alkaline hydrolysis, H_2_O_2_ oxidation, US treatment	NC grafted with maleic anhydride and styrene, partial substitute of CB	SBR	RT mechanical mixing	35 phr CB + 10 phr m-CNC	From 30 to 32.5	From 590 to 600	M 300	From 15.5 to 16.0	Not reported	[128]
CNC	Cotton linter	HCl hydrolysis	Antioxidant gallic acid added to CNC	NBR	RT mechanical mixing	3 phr m-CNC	From 8.26 to 11.86	From 3.46 to 4.20	M 100	From 1.06 to 1.19	Not reported	[156]
CNC	Cotton linter	HCl hydrolysis	Acetic anhydride added to CNC	NBR	RT mechanical mixing	5 phr m-CNC	From 8.264 to 16.228	From 3.257 to 4.171	M 100	From 15.008 to 19.388	Not reported	[157]
CNC	n/a	Without treatment	n/a	NBR	US treatment, RT mechanical mixing	3 phr CNC, 2 days maturation	From 4.3 to 11.5	From 145 to 167	YM	From 2.5 to 130	From 0.5 to 2.4 J/m^3^	[158]
CNC	n/a	Without treatment	n/a	CR	Homogenization, US treatment, RT mechanical mixing	3 wt % CNC	From 4.7 to 5.8	From 846 to 660	YM	From 1.3 to 12.5	Not reported	[159]
			CNC grafted with PLA			3 wt % m-CNC	From 4.7 to 6.3	From 846 to 640		From 1.3 to 19.9		
CNC	Cotton	Without treatment	n/a	BR	US treatment, RT mechanical mixing	10 wt % CNC	From 0.31 ± 0.01 to 0.39 ± 0.07	From 515 ± 35 to 492 ± 27	YM	From 0.7 ± 0.1 to 0.9 ± 0.2	Not reported	[160]
			BR partially modified with adamantane			10 wt % CNC	From 0.24 ± 0.04 to 0.40 ± 0.05	From 420 ± 30 to 390 ± 19		From 0.8 ± 0.1 to 1.1 ± 0.3		
			BR modified with adamantane and β-cyclodextrin			10 wt % β-CD + 15 wt % CNC	From 1.51 ± 0.02 to 3.43 ± 0.06	From 341 ± 24 to 103 ± 7		From 2.2 ± 0.2 to 6.9 ± 0.4		
CNC	Chili (*Capsicum annum*)	Alkali treatment, bleaching, H_2_SO_4_ hydrolysis	n/a	IR	RT mechanical mixing	6 wt % CNC	From 0.202 ± 0.010 to 0.359 ± 0.040	Not reported	YM	From 4.8 ± 1.1 to 24.6 ± 3.0	Not reported	[161]
CNF	n/a	Without treatment	n/a	SBR	RT homogenization and blending	7 phr CNF	From 3.20 ± 0.71 to 8.06 ± 0.95	From 714 ± 131 to 786 ± 88	YM	From 1.67 ± 0.06 to 10.33 ± 1.73	Not reported	[25]
			CNF surface modified with 3-mercaptopropanoic acid			7 phr m-CNF	From 3.20 ± 0.71 to 9.03 ± 0.29	From 714 ± 131 to 357 ± 48		From 1.67 ± 0.06 to 9.78 ± 1.95		
			CNF surface modified with 11-mercaptoundecanoic acid			7 phr m-CNF	From 3.20 ± 0.71 to 8.66 ± 0.57	From 714 ± 131 to 204 ± 30		From 1.67 ± 0.06 to 11.51 ± 1.76		
			CNF surface modified with 4-pentanoic acid			9 phr m-CNF	From 3.20 ± 0.71 to 12.77 ± 0.16	From 714 ± 131 to 157 ± 16		From 1.67 ± 0.06 to 22.14 ± 2.78		
			CNF surface modified with 10-undecenoic acid			7 phr m-CNF	From 3.20 ± 0.71 to 9.37 ± 0.34	From 714 ± 131 to 332 ± 12		From 1.67 ± 0.06 to 9.67 ± 1.18		
			CNF surface modified with cysteine			9 phr m-CNF	From 3.20 ± 0.71 to 10.32 ± 0.39	From 714 ± 131 to 276 ± 14		From 1.67 ± 0.06 to 12.79 ± 1.58		
CNF	Hardwood pulp	HP homogenization, solvent exchange	CNF surface modified with DTACl	SBR	Mechanical mixing at 100 °C	10 wt % m-CNF	From 10 ± 1 to 55 ± 4	From 368 ± 25 to 406 ± 13	YM	From 2.0 ± 0.1 to 6.8 ± 0.3	Not reported	[26]
CNF	Wood	TEMPO- oxidation	Solvent exchange for getting a homogenous system with N, N-dimethylformamide (DMF) solution	H-NBR	RT mechanical mixing	5 wt % m-CNF	From 4.8 to 17.5	From 750 to 490	YM	From 4 to 46	From 22.0 to 51.0 MJ/cm^3^	[162]
CNF	Kenaf fiber	Mercerization, bleaching and sonication	IR/PLA compound blend	IR/PLA	Melt compounding technique	3 wt % CNF + 10 wt % IR + 87 wt % PLA	Negligible change at 60	Not reported	YM	From 600 to 1180	Not reported	[163]
BC	*Acetobacter xylinum*	H_2_SO_4_ hydrolysis	n/a	XNBR	US treatment	13 phr BC	From 2.90 ± 0.32 to 12.21 ± 0.10	From 228 ± 30 to 195 ± 10	YM	From 1.7 ± 0.2 to 4.3 ± 0.1	Not reported	[129]

^1^ Reference samples to represent the studies are those that provide the greatest improvement in tensile strength. Elongation at break, modulus and strain energy density values correspond to that sample. ^2^ The types of moduli reported were classified accordingly as M (not explicitly defined), YM (Young’s modulus) and Mx (where x is a specific strain value). ** Standard deviation and significant digits are reported as-is. Value approximations are made if data charts are provided without numerical values.

## Data Availability

Not applicable.
